# Recombinant Murine Gamma Herpesvirus 68 Carrying KSHV G Protein-Coupled Receptor Induces Angiogenic Lesions in Mice

**DOI:** 10.1371/journal.ppat.1005001

**Published:** 2015-06-24

**Authors:** Junjie Zhang, Lining Zhu, Xiaolu Lu, Emily R. Feldman, Lisa R. Keyes, Yi Wang, Hui Fan, Hao Feng, Zanxian Xia, Jiya Sun, Taijiao Jiang, Shou-jiang Gao, Scott A. Tibbetts, Pinghui Feng

**Affiliations:** 1 Department of Molecular Microbiology and Immunology, Norris Comprehensive Cancer Center, Keck School of Medicine, University of Southern California, Los Angeles, California, United States of America; 2 Department of Molecular Genetics and Microbiology, University of Florida, Gainsville, Florida, United States of America; 3 College of Life Sciences, Hunan Normal University, Changsha, Hunan, China; 4 State Key Laboratory of Medical Genetics and School of Life Sciences, Central South University, Changsha, Hunan, China; 5 Center for Systems Medicine, Institute of Basic Medical Sciences, Chinese Academy of Medical Sciences & Peking Union Medical College, Beijing; Suzhou Institute of Systems Medicine, Suzhou, China; 6 Key Laboratory of Protein and Peptide Pharmaceuticals, Institute of Biophysics, Chinese Academy of Sciences, Beijing, China; Louisiana State University Health Sciences Center, UNITED STATES

## Abstract

Human gamma herpesviruses, including Kaposi’s sarcoma-associated herpesvirus (KSHV) and Epstein-Barr virus (EBV), are capable of inducing tumors, particularly in in immune-compromised individuals. Due to the stringent host tropism, rodents are resistant to infection by human gamma herpesviruses, creating a significant barrier for the *in vivo* study of viral genes that contribute to tumorigenesis. The closely-related murine gamma herpesvirus 68 (γHV68) efficiently infects laboratory mouse strains and establishes robust persistent infection without causing apparent disease. Here, we report that a recombinant γHV68 carrying the KSHV G protein-coupled receptor (kGPCR) in place of its murine counterpart induces angiogenic tumors in infected mice. Although viral GPCRs are conserved in all gamma herpesviruses, kGPCR potently activated downstream signaling and induced tumor formation in nude mouse, whereas γHV68 GPCR failed to do so. Recombinant γHV68 carrying kGPCR demonstrated more robust lytic replication *ex vivo* than wild-type γHV68, although both viruses underwent similar acute and latent infection *in vivo*. Infection of immunosuppressed mice with γHV68 carrying kGPCR, but not wild-type γHV68, induced tumors in mice that exhibited angiogenic and inflammatory features shared with human Kaposi’s sarcoma. Immunohistochemistry staining identified abundant latently-infected cells and a small number of cells supporting lytic replication in tumor tissue. Thus, mouse infection with a recombinant γHV68 carrying kGPCR provides a useful small animal model for tumorigenesis induced by a human gamma herpesvirus gene in the setting of a natural course of infection.

## Author Summary

Human gamma herpesviruses, including Epstein-Barr virus (EBV) and Kaposi’s sarcoma-associated herpesvirus (KSHV), are causatively linked to a spectrum of human oncogenic malignancies. Due to the stringent host restriction, rodents are generally not amenable to infection by EBV and KSHV. Murine gamma herpesvirus 68 (γHV68) is closely related to KSHV and EBV, although infection in mouse does not manifest apparent diseases. Here we developed a recombinant γHV68 that carries the KSHV G protein-coupled receptor, an important signaling molecule implicated in KSHV pathogenesis. Intriguingly, laboratory mice infected with this recombinant γHV68 developed angiogenic lesions that resembled human Kaposi’s sarcoma. This mouse infection with recombinant γHV68 carrying KSHV GPCR represents a useful model to investigate viral oncogenesis induced by human gamma herpesvirus in the context of viral infection.

## Introduction

Herpesviruses are ubiquitous pathogens in humans that have been implicated in an array of human diseases, including a wide range of cancers. The human gamma herpesviruses, including Epstein-Barr virus (EBV) and Kaposi’s sarcoma-associated herpesvirus (KSHV), are capable of promoting tumor formation in immune-compromised individuals, e.g., human immunodeficiency virus (HIV)-infected patients and organ transplant recipients [1–4]. KSHV is the etiological agent of human Kaposi’s sarcoma (KS), primary effusion lymphoma and multicentric Castleman’s disease (MCD) [1–4]. KS is the most common neoplasm in AIDS patients that manifests in the skin, the surface of internal organs and the oral cavity. Particularly, the oral cavity is an important compartment for KSHV infection, replication and dissemination. Histologically, KS tumor lesions are composed of both latently-infected spindle cells and a small subset of cells supporting lytic replication of KSHV [5–7]. The prevailing postulate is that these two types of infection programs synergize in fostering the genesis of sarcoma or lympho-proliferative disorders [8].

In the presence of an active immune response, human EBV and KSHV have evolved to establish a lifelong persistent infection. To date, KSHV has been reported to infect only primates [9], demonstrating a narrow host range for successful de novo infection in vivo [10,11]. Thus, most pathogenesis studies of human gamma herpesvirus genes involving small rodents rely heavily on transgenic or xenograft nude mouse models. For example, in transgenic models, the KSHV gene products LANA and vFLIP induce lymphoproliferative diseases that display some pathological features similar to human PEL or, to a less extent, MCD [12,13]. In contrast, mice with endothelium- or other tissue-specific expression of kGPCR, developed angiogenic lesions in the skin that resemble human KS [14–16]. These models provide important tools to investigate the interaction between viral transforming proteins and key cellular signaling pathways in instigating tumor formation; however, these studies take place outside of the context of viral infection of a natural host. Presumably, infection of a co-evolved host will provide additional layers of interaction between viral factors and host signaling components. Expression of multiple viral genes also triggers complex immune responses that likely influence the course and outcome of disease.

Murine gamma herpesvirus 68 (γHV68) is genetically related to human KSHV and EBV [17,18]. γHV68 infection results in a transient lytic replication in the lung or spleen that is followed by persistent infection, primarily in the form of latency [19]. γHV68 infection causes no apparent symptoms in immune-competent laboratory mouse strains, but lymphoproliferative disease and B cell lymphoma have been reported in β2-microglobulin-deficient mice that lack CD8 T cells [20]. Genome sequencing and functional studies have pointed to a number of viral factors that collectively contribute to the pathogenesis of KSHV. In the KSHV genome, genes encoding viral factors cluster into the K3-K7 locus and the latency locus (including ORF74) are implicated in KSHV pathogenesis, notably tumorigenesis. One interesting example is the viral G protein-coupled receptor, kGPCR (also known as ORF74) [21]. kGPCR is a homologue of the human IL-8 receptor and constitutively activates downstream signaling cascades without its cognate ligands. Compared to the IL-8R, kGPCR binds to a wide spectrum of chemokines that can alter kGPCR-mediated signal transduction [22–26]. In the absence of cognate ligand, kGPCR constitutively activates downstream signaling. All gamma herpesviruses encode a GPCR in their genomes, despite the functionality of these viral GPCRs may differ. The γHV68 mGPCR demonstrated no basal level of signaling activity that was only detected upon ligand stimulation, behaving like a cellular GPCR in signal transduction [27]. mGPCR-mediated signaling was proposed to promote viral lytic replication [27].

Human gamma herpesviruses demonstrate restricted host range and the lack of animal models hinders the in vivo study on these important human pathogens. We report here that introduction of kGPCR into the γHV68 genome rendered γHV68 the ability to induce tumor formation. Histologically, tumors derived from mice infected with recombinant γHV68.kGPCR consisted of spindle-shaped cells and significant infiltrated immune cells. These signature components recapitulate key pathological features of human KS tumors. Importantly, cells with latent infection and lytic replication were detected in tumor tissues. Thus, we have engineered a recombinant γHV68 that is capable of inducing tumor formation in mice, providing a valuable tool to investigate tumorigenesis in the context of viral infection of a natural host.

## Results

### kGPCR, but not γHV68 GPCR (mGPCR), is constitutively active

**Fig 1:**
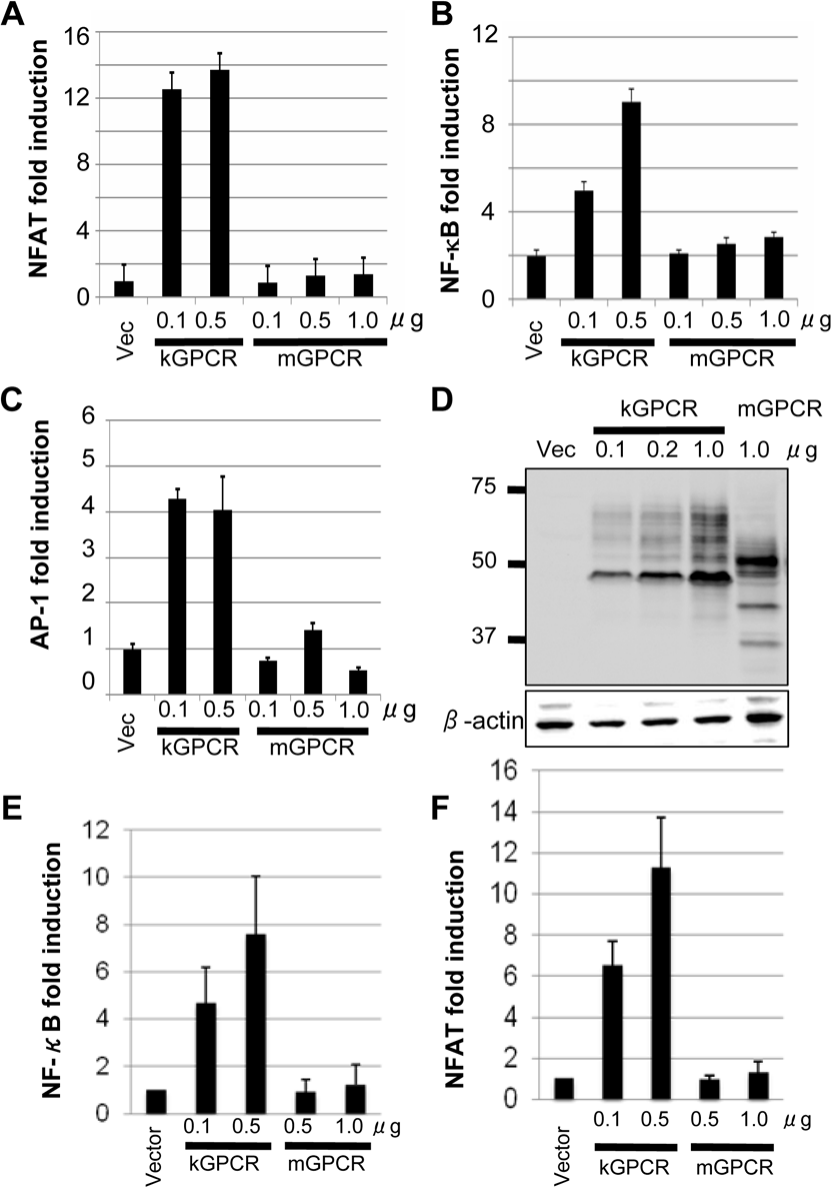
KSHV GPCR (kGPCR), but not murine gamma herpesvirus 68 GPCR (mGPCR), activates downstream signaling events. 293T cells were transfected with a reporter plasmid cocktail of NFAT (A and E), NF-κB (B and F) and AP-1 (C), and increasing amount of a plasmid containing either kGPCR or mGPCR. Reporter luciferase assays were performed at 30 hours post-transfection with whole cell lysates to determine activation of NFAT, NF-κB and AP-1. (D) 293T cells were transfected with increasing amount of a plasmid containing kGPCR or mGPCR. Whole cell lysates were analyzed by immunoblotting with anti-HA and anti-actin antibodies. (E and F) NIH 3T3 cells were transfected with NF-κB (E) and NFAT (F) reporter cocktail as in (A), except with lipofectamine. Activation of NF-κB and NFAT was determined by luciferase reporter assays.

The KSHV kGPCR (ORF74) is a constitutively active signaling molecule, independent of ligand binding [22]. In contrast, the γHV68 mGPCR requires ligand binding to induce downstream signaling [27]. We thus compared the signaling capacity of kGPCR and mGPCR. Previous studies have shown that kGPCR activates signaling events that, ultimately, culminate in up-regulating gene expression driven by NF-κB, NFAT and AP-1 transcription factors [28–31]. We utilized luciferase reporter assays to probe the activation of NF-κB, NFAT and AP-1 by kGPCR and mGPCR. In transfected 293T cells, kGPCR expression highly elevated the expression of an NFAT-dependent reporter (Fig 1A). By contrast, mGPCR expression had no detectable effect under the same conditions. kGPCR, but not mGPCR, modestly up-regulated gene expression driven by responsive elements of NF-κB and AP-1 transcription factors (Fig 1B and 1C). Immunoblotting analysis showed that mGPCR and kGPCR were expressed at comparable levels in 293T cells (Fig 1D). Similar results were obtained from murine NIH 3T3 fibroblasts that support robust lytic replication of γHV68, i.e., kGPCR activated NF-κB and NFAT-dependent gene expression, but mGPCR failed to do so (Fig 1E and 1F). Collectively, these results show that kGPCR, but not mGPCR, activates NF-κB, NFAT and AP-1 transcription factors, signaling events underpinning kGPCR tumorigenesis.

### kGPCR, but not mGPCR, activates AKT and induces tumor formation

**Fig 2:**
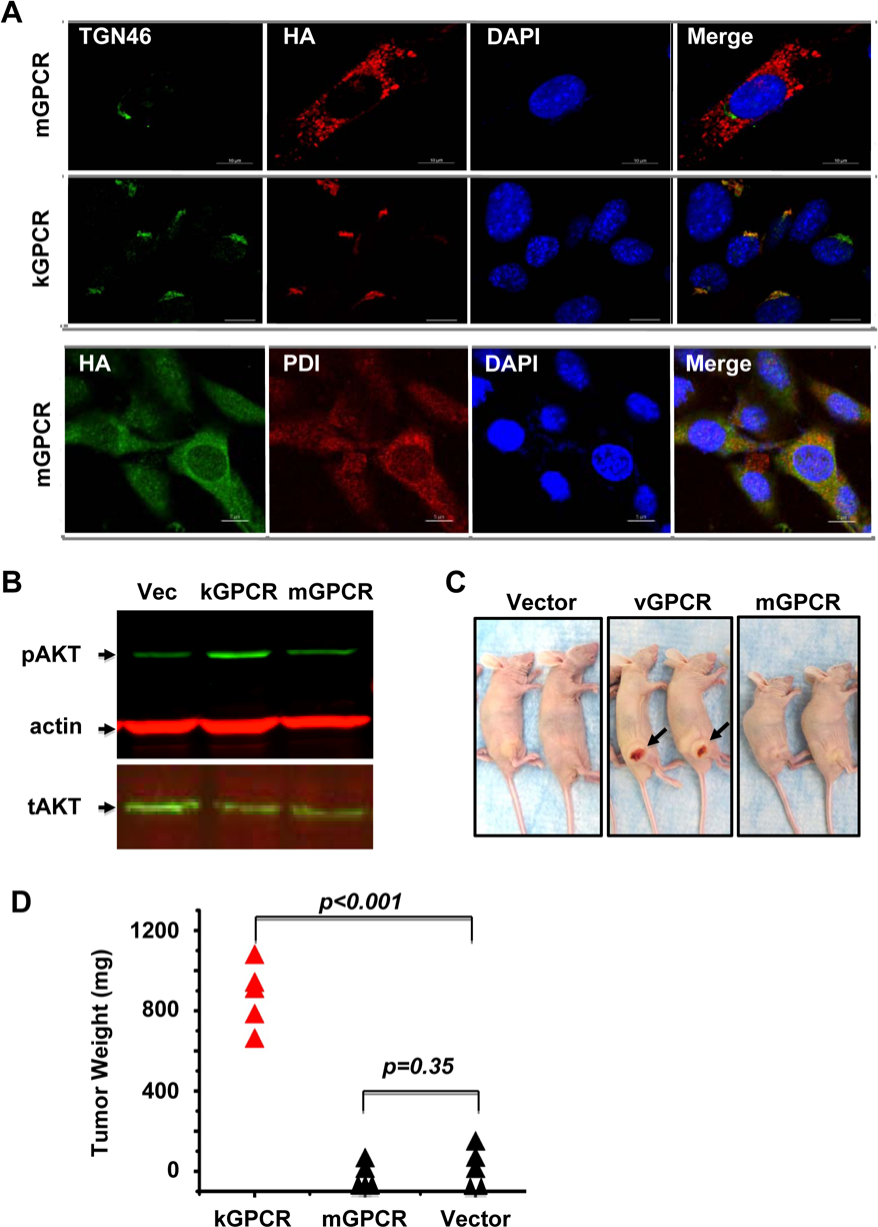
Differential signaling and tumorigenic capacity of kGPCR and mGPCR. (A) Mouse SVEC endothelial cells stably expressing kGPCR or mGPCR were fixed and stained with antibodies against the HA epitope (kGPCR or mGPCR), TGN46 (Golgi marker) or PDI (ER marker) and nuclei were stained with DAPI. Representative images were collected with Nikon and processed with ImageJ. Scale bar denotes 10 μm. (B) Control SVEC or SVEC stable cells expressing kGPCR or mGPCR were starved overnight and whole cell lysates were analyzed with indicated antibodies. pAKT and tAKT indicate phosphorylated and total AKT, respectively. (C) and (D) Control SVEC or SVEC stable cells expressing kGPCR or mGPCR (5 × 10^5^), along with 1 × 10^6^ bystander regular SVEC cells, were injected into the flank of nude mice. Tumors were photographed at four weeks post-injection (C) and tumor weight was determined (D).

Cellular and viral GPCRs primarily localize to the cell surface where extracellular stimuli regulate intracellular signal transduction. kGPCR is unique in that it is predominantly retained in the trans-golgi network (TGN), although its cell surface expression can be detected as well [30]. To compare the intracellular localization of kGPCR and mGPCR, we established mouse SVEC endothelial cells that stably express kGPCR and mGPCR with lentiviral infection. Confocal immunofluorescence microscopy analysis showed that kGPCR resided in the TGN, co-localizing with TGN46 (Fig 2A). By contrast, mGPCR was dispersed in the cytoplasm, displaying an intracellular localization distinct from TGN46 staining. The subcellular pattern of mGPCR was reminiscent of the endoplasmic reticulum (ER) compartment (Fig 2A). Indeed, mGPCR co-localized with protein disulfide isomerase, an ER resident protein, as analyzed by confocal microscopy (Fig 2A). These results indicate that kGPCR and mGPCR reside in the TGN and ER compartment, respectively. However, we would like to point out that the cell surface expression of both viral GPCRs is expected.

Signaling pathways that are constitutively activated by kGPCR, including the PI3K-AKT cascade that promotes cell proliferation and survival, underpin the tumorigenesis of kGPCR [14,32,33]. We thus compared the effect of kGPCR and mGPCR on AKT activation. Stable expression of kGPCR in SVEC cells elevated AKT phosphorylation at serine 437 under starvation condition, indicative of AKT activation (Fig 2B). mGPCR expression in SVEC cells did not significantly impact the level of phosphorylated AKT under similar conditions.

To compare the tumorigenic potential of kGPCR and mGPCR, we initially utilized a xenograft nude mouse model. In this model, SVEC cells stably expressing kGPCR or mGPCR were mixed with bystander regular SVEC cells to assess the autocrine and paracrine action of kGPCR in promoting tumor formation. At two weeks post-injection, tumors were detected in mice that were grafted with SVEC cells expressing kGPCR. No tumors were detected in mice that were grafted with control SVEC cells or SVEC cells expressing mGPCR, even at four weeks post-inoculation when mice grafted with kGPCR-expressing SVEC cells had to be euthanized (Fig 2C). Tumors derived from SVEC cells expressing kGPCR were highly vascularized, which was visible in the skin, and tumor weight averaged ~850 mg (Fig 2D). Collectively, these results indicate that kGPCR, but not mGPCR, potently activates signaling events and induces tumor formation derived from murine SVEC endothelial cells.

### Generation of recombinant γHV68 that carries kGPCR at the mGPCR locus

**Fig 3:**
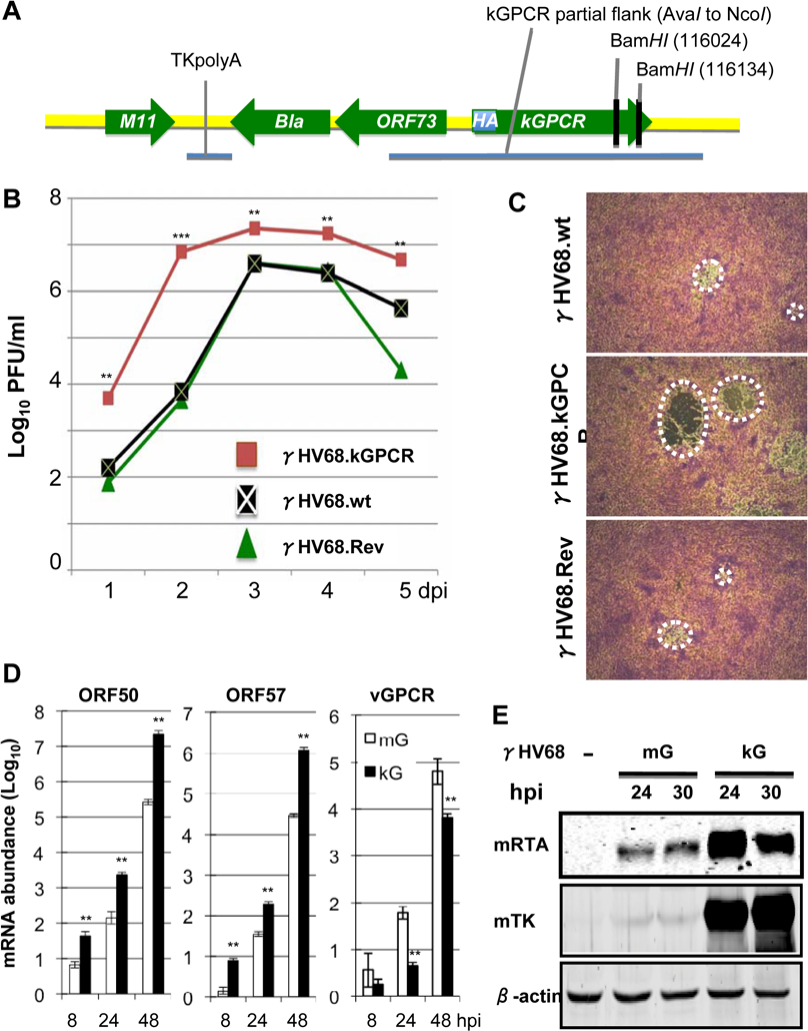
Generation and characterization of a recombinant γHV68 carrying kGPCR. (A) Diagram of the GPCR locus, in relation to the inserted *Bla* gene (encoding beta-lactamase), of the recombinant γHV68.kGPCR, wherein mGPCR was replaced with kGPCR carrying an upstream HA epitope. (B) and (C) NIH 3T3 cells were infected with recombinant γHV68.wt, γHV68.kGPCR or γHV68 revertant (γHV68.Rev) (MOI = 0.05) and whole cell lysates were prepared with three times of freeze/thaw. Infectious unit of γHV68 was determined by plaque assay using NIH 3T3 monolayer (B) and plaques were photographed (C). (D) and (E) NIH 3T3 were infected with recombinant γHV68 as indicated. Cells were harvested at indicated time points post-infection. Extracted total RNA was analyzed by reverse transcription and real-time PCR with primers specific for indicated genes (D). Whole cell lysates were prepared and analyzed by immunoblotting with antibodies against RTA, thymidine kinase (TK or ORF21) and β-actin (E). For (B) and (D), p values were calculated in reference to the control γHV68 wild-type group, *p < 0.05; **p < 0.01; ***p < 0.001.

The cell proliferative and tumorigenic potential of kGPCR, compared to mGPCR, prompted us to engineer a recombinant γHV68 that expresses kGPCR in place of mGPCR and under control of the endogenous mGPCR promoter. To facilitate detection of kGPCR, we also inserted an HA epitope immediately after the start codon of kGPCR (Fig 3A). The GEMBO Bacmid, which incorporates the beta-lactamase (*Bla*) marker gene at the latent locus was selected as the parental backbone to introduce kGPCR into the mGPCR locus [34]. Viruses produced from the GEMBO BACmid were used to mark cells that are latently infected by γHV68. Once recombinant γHV68 that carries kGPCR (designated γHV68.kGPCR) was generated, we also introduced mGPCR back to the same locus to replace the HA.kGPCR, yielding γHV68 revertant (γHV68.rev). BAC DNA was purified from bacteria and digested with Bam*HI* to analyze the overall digestion pattern. Due to two Bam*HI* sites located at the 3’ end of kGPCR, a ~5.3 kb fragment disappeared in γHV68.kGPCR BAC DNA, presumably Bam*HI* digestion converted the ~5.3 kb fragment into a ~5 kb fragment that already existed (S1A Fig). The kGPCR locus was further PCR amplified and sequenced, which validated the introduction of the HA-tagged kGPCR, without any additional mutations (S1B Fig). Furthermore, we employed genome sequencing to compare the BAC DNA of γHV68 wild-type with that of γHV68.kGPCR. This identified three point mutations within γHV68.kGPCR, excluding the difference in kGPCR (S1C Fig). Taken together, the sequencing data indicate the overall integrity of these recombinant γHV68 genomes.

BAC DNA was then transfected into NIH 3T3 cells to produce recombinant γHV68. Recombinant γHV68 viruses were passaged in NIH 3T3/Cre cells three times to remove the backbone of γHV68 BAC DNA and plaque assays were performed to determine the titer of recombinant γHV68. Multi-step growth of recombinant γHV68 indicated that wild-type γHV68 and γHV68.rev replicated indistinguishably in NIH 3T3 cells. Interestingly, γHV68.kGPCR replicated with much faster kinetics and reached higher titer in NIH 3T3 cells, compared to wild-type γHV68 (Fig 3B). This result indicates that kGPCR can enhance γHV68 replication when expressed from infected cells. Additionally, γHV68.kGPCR consistently formed larger plaques in NIH 3T3 cells than wild-type γHV68 or γHV68.rev (Fig 3C). The larger plaque sizes did not correlate with more robust syncitia. Thus, the increased replication of γHV68.kGPCR contributes to the larger plaques. Real-time PCR analysis with primers specific for viral lytic genes indicated that higher levels of expression of lytic genes in cells infected with γHV68.kGPCR than those of cells infected with wild-type γHV68 (Figs 3D and S1D). Surprisingly, kGPCR mRNA level was significantly lower than that of mGPCR at all time points post-infection (Fig 3D). Immunoblot analysis further confirmed more robust viral RTA and TK expression in cells infected with γHV68.kGPCR than those in cells infected with wild-type γHV68 (Fig 3E). Taken together, these results indicate that kGPCR expression can promote γHV68 lytic replication.

### Lytic replication and latent infection of γHV68.kGPCR in vivo

**Fig 4:**
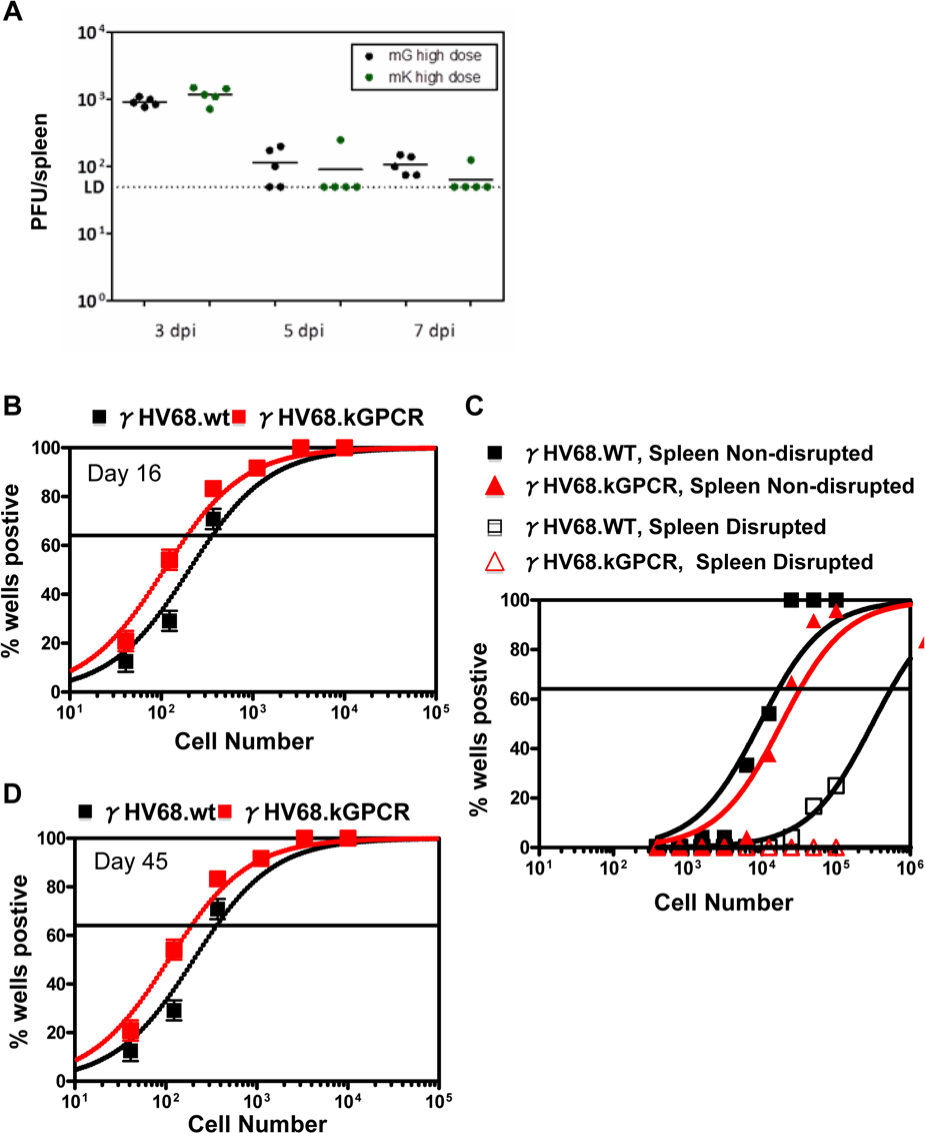
Acute and latent infection of γHV68 in mouse. Balb/c mice, sex- and age-matched, were infected with wild-type γHV68 (mG) or γHV68.kGPCR (mK) (1 × 10^5^ pfu) via intraperitoneal injection. Spleens were collected at 3, 5, and 7 days post-infection (dpi) and viral titer was determined by plaque assay (A). Alternatively, spleens were collected at 16 (B) and 45 dpi (D). Viral episomal genome was determined by limiting-dilution PCR analysis. (C) Splenocytes, either intact or disrupted with mechanic force, were co-cultured with mouse embryonic fibroblasts to allow plaque formation for 21 days.

Considering that γHV68.kGPCR replicated more robustly than wild-type γHV68 in vitro, we examined the lytic replication during acute infection of BALB/c mice, a wild-type inbred strain amenable to tumor development. When BALB/c mice were infected with high dose of virus (1 × 10^5^ pfu) via intra-peritoneal injection, we found that approximately 1 × 10^3^ pfu per spleen was detected by plaque assay at 3 days post-infection (dpi) for both wild-type γHV68 and γHV68.kGPCR, indicating significant lytic replication (Fig 4A). At 5 and 7 dpi, γHV68 replication was about 1 × 10^2^ pfu per spleen. At all three time points, similar levels of viral loads were detected in mice infected with wild-type γHV68 and γHV68.kGPCR. None of the infected mice showed disease symptoms at the time of harvest.

Regardless of the route and dose of infection, γHV68 efficiently establishes latent infection in splenocytes, including B lymphoid cells and other immune cells (e.g., macrophages) [35]. We examined the latent infection in splenocytes of BALB/c infected with recombinant wild-type γHV68 and γHV68.kGPCR. Limiting-dilution PCR analysis indicated that the frequency of splenocytes carrying γHV68.kGPCR was ~2-fold higher than that of wild-type γHV68, approximately one in 100 cells at 16 days post-infection (Fig 4B). However, this was not due to increased frequencies of reactivation or preformed virus, as parallel samples of splenocytes from γHV68.kGPCR-infected mice exhibited slightly lower levels of both compared to wild-type γHV68-infected mice (Fig 4C). Furthermore, wild-type γHV68- and γHV68.kGPCR-infected mice exhibited similarly lower frequencies of infection at 45 days post-infection (Fig 4D), while reactivation and preformed virus were below the detection limit (S2 Fig). These results show that wild-type γHV68 and γHV68.kGPCR establish similar levels of latent infection in splenocytes in vivo.

### Recombinant γHV68.kGPCR induces angiogenic lesions in mouse

**Fig 5:**
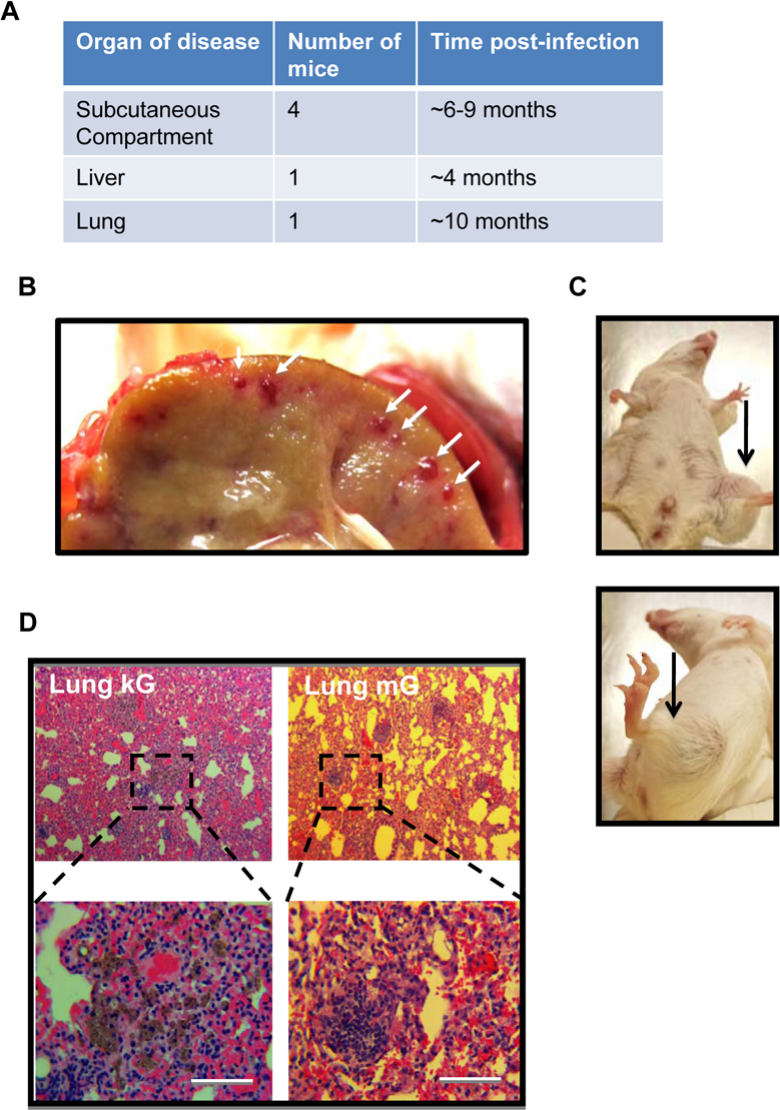
γHV68.kGPCR infection induces angiogenic lesions in immune-suppressed mouse. Balb/c mice, of six to eight-week-old females, were infected with (1 × 10^5^ pfu) via intraperitoneal injection. (A) Summary of 5 mice, out of 15, that demonstrated diseased conditions. One mouse had both subcutaneous tumor and lung fibrosis (shown in D). Mouse liver carrying angiogenic nodule (B) and subcutaneous tumors within the hinder legs (C) were photographed. (D) Lung lesions of mice infected with γHV68.kGPCR and wild-type γHV68 analyzed by H&E staining.

Having characterized lytic replication and latent infection of recombinant γHV68.kGPCR in mice, we next assessed whether infection by γHV68.kGPCR can induce tumors in BALB/c mice. In a pilot experiment, we set up two groups of mice that were infected with either wild-type γHV68 or γHV68.kGPCR. At three weeks post-infection, mice were injected with cyclosporine A (CsA) to inhibit T cell response. Out of a total of 15 experimental mice that were infected with γHV68.kGPCR and treated with CsA, five mice displayed malignant conditions of the liver, lung and subcutaneous compartment (Fig 5A). Over the course of six months of the experiment, one out of five mice that were infected with γHV68.kGPCR and treated with CsA died. Although the mouse had no apparent lesion on the skin, inspection of its internal organs identified highly vascular nodules in the liver (Fig 5B). The reddish lesions of apparent size share similar characteristics of highly vascularized lesions with human nodular KS. Another mouse demonstrated localized fibrosis in the lung (Fig 5C), which displayed none of the angiogenic characteristics of human Kaposi’s sarcoma.

**Fig 6:**
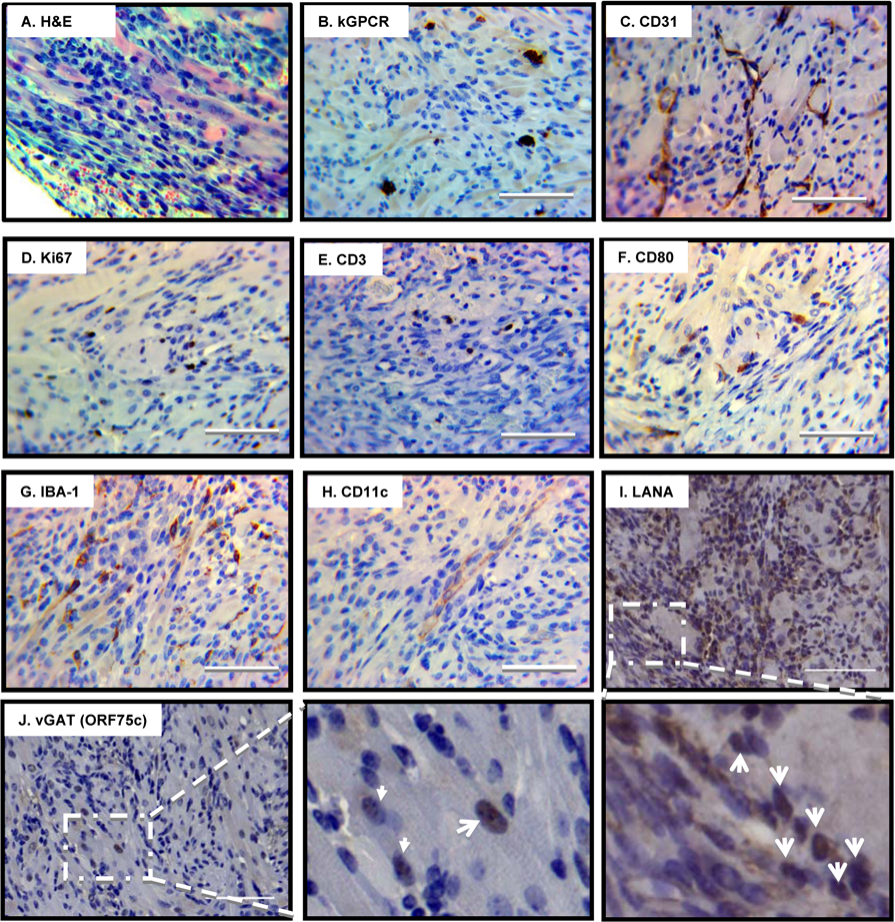
Pathological analysis of angiogenic tumors derived from γHV68.kGPCR-infected mice. Subcutaneous tumors developed in γHV68.kGPCR-infected BALB/c mice were analyzed by hematoxylin & eosin (H&E) staining (A) and immunohistochemistry staining with antibodies against the HA epitope (kGPCR, B), endothelial marker CD31 (C), proliferation marker Ki67 (D), T cell marker CD3 (E), CD80 (F), macrophage marker IBA-1 (G) and dendritic cell marker CD11c (H). Tumors were also analyzed by immunohistochemistry staining with antibodies against γHV68 LANA (ORF72, latent antigen) (I) and vGAT (ORF75c, lytic antigen) (J). Boxed regions were amplified right below (I) or next (J) to the original images. Scale bars denote 25 μm.

In addition to the hepatic KS-like lesion observed in one mouse, 4 mice developed large subcutaneous tumors in a hind leg at 5 months post-infection with γHV68.kGPCR and treated with CsA, although no apparent skin lesion was observed (Fig 5D). The tumor was large and highly vascular, consistent with the notion that kGPCR induces angiogenesis of endothelial cells. H&E staining showed that the tumor cells were spindle-shaped and packed into slit-like structure, infiltrated with large numbers of erythrocytes (Fig 6A). Large numbers of immune cells were also observed within or proximal to the highly packed tumor region. IHC staining revealed that kGPCR was expressed in a small subset of cells scattered in the tumor lesion, which is consistent with the paracrine action of kGPCR in human KS tumors (Figs 6B and S3A). Given the suspected origin of endothelial cells of KS, we examined the tumor tissue with antibodies against an endothelial marker CD31 and found that a significant portion of tumor cells expressed high levels of CD31, most of which were elongated and spindle-shaped cells (Figs 6C and S3B). These cells appeared to connect with each other, implying that they were undergoing proliferation in these tumors. Indeed, a significant fraction of tumor cells were stained positive for Ki67 (Figs 6D and S3C), a marker of proliferating cells.

Human KS lesions demonstrate hallmarks of excessive inflammatory response, with infiltration of a wide range of immune cells. Thus, we determined whether immune cells are present in the angiogenic lesions that developed in mice infected with γHV68.kGPCR. To this end, we performed IHC to probe T cells, macrophages and dendritic cells. Antibody against human CD3 identified scarce T cells in the tumor lesion (Fig 6E). The low level of T cells is likely due to the immune suppressive effect of cyclosporine A. To determine whether this is due to lack of antigen education, we further probed antigen-presenting cells with antibody against CD80 (also known as B7-1) that provides co-stimulatory signal for T cell activation. CD80 is generally expressed on the cell surface of activated B cells and monocytes [36], classic antigen-presenting cells. Interestingly, a small number of cells were positively stained with anti-CD80 antibodies and these cells had a small cytoplasm, morphological characteristics of B cells and monocytes (Fig 6F). Finally, we examined the relative frequency of macrophages and dendritic cells in the tumor lesions. These innate immune cells have been implicated in antigen presentation and cytokine production, thus influencing the course of tumorigenesis. When tumor tissues were stained with antibodies against IBA-1 and CD11c, markers for macrophages and dendritic cells. We found that large number of cells were positive for IBA-1 and well distributed in the tumor lesion (Figs 6G and S3D). By contrast, cells stained for CD11c were much less and localized (Fig 6H). These cells demonstrated elongated and spindle-shaped cytoplasm, forming the slit-like structure within tumors. Overall, these H&E staining and IHC analyses demonstrate that the tumor derived from γHV68.kGPCR virus displayed significant number of immune infiltrates, signature inflammatory components of human KS.

An important characteristic of human KS is the presence of predominant KSHV latently-infected cells and low percentage of cells supporting lytic replication of KSHV. It was postulated that a synergy between cells of latent and lytic KSHV programs fuels the sarcomagenesis. We first examined the latently infected cells with an antibody against the latency-associated nuclear antigen (LANA), a hallmark antigen for latent γHV68 infection. Using mock- and γHV68-infected NIH 3T3 cells, we showed that the purified rabbit serum was highly specific to LANA antigen by immunoblotting and immunofluorescence staining (S4A and S4B Fig). When tumor tissues were analyzed by IHC staining, we observed that approximately 20–30% of tumor cells were positive for LANA expression in the nucleus (Fig 6I). LANA positive cells were more likely present in the highly packed slit-like structure. To detect cells supporting γHV68 lytic replication, we analyzed tumor tissues by IHC with an antibody against vGAT (ORF75c), a tegument protein of virion particle. Immunoblotting and immunofluorescence staining demonstrated that the purified anti-vGAT antibody was highly specific for vGAT in γHV68-infected NIH 3T3 cells (S4C and S4D Fig). As shown in Fig 6J, vGAT-positive cells were apparent in the tumor section and accounted for about 2–3% of tumor cells. The frequency of vGAT-positive cells is similar to that of kGPCR-expressing cells (Fig 6B). Thus, similar to KS lesions, cells of latency and lytic replication programs of γHV68 are present in tumor tissue derived from γHV68.kGPCR-infected mice.

We used cyclosporine A to suppress host T cell immunity, which facilitated tumor formation in mice infected with γHV68.kGPCR. Cyclosporine A is a potent inhibitor of NFAT activation and kGPCR activates NFAT. Thus, we examined the effect of cyclosporine A on the lytic replication of γHV68.kGPCR and wild-type γHV68. The latter serves as a control for γHV68.kGPCR. However, cyclosporine A had no significant effect on viral replication in NIH 3T3 cells as determined by plaque assay (S5A–S5D Fig). Thus, cyclosporine does not intrinsically promote the lytic replication of γHV68.kGPCR in cultured cells.

### Histopathological features of human Kaposi’s sarcoma

**Fig 7:**
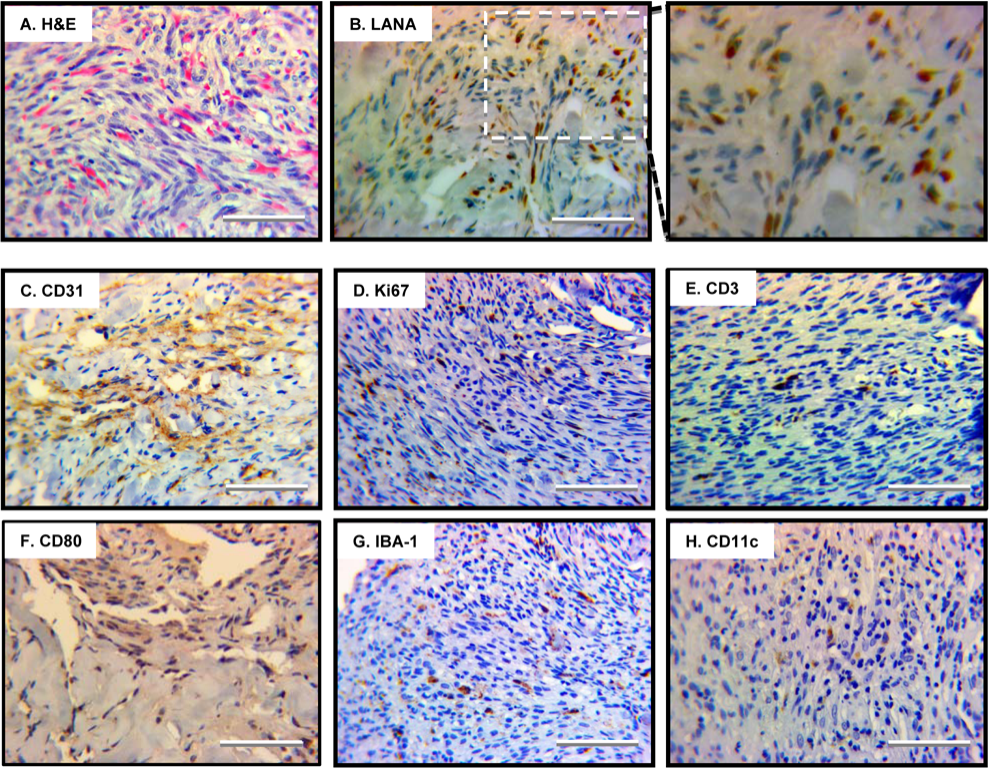
Angiogenic and inflammatory features of human Kaposi’s sarcoma. Human Kaposi’s sarcoma lesion was analyzed by H&E staining (A) and immunohistochemistry staining with antibodies against the latent nuclear antigen LANA (B), markers for endothelial cells CD31 (C), proliferating cells Ki67 (D), T cells CD3 (E), antigen-presenting cells CD80 (F), macrophage IBA-1 (G) and dendritic cells CD11c (H). Representative images were shown and scale bars denote 25 μm.

To compare the tumors derived from mice infected with γHV68.kGPCR to human KS, we examined the inflammatory cells present in human KS samples that have been previously reported [37]. H&E staining revealed that the tumor tissue was filled with a large number of erythrocytes, which were widely distributed within the tumor lesion (Fig 7A). Tumor cells of irregular nuclei were abundant and packed into slit-like structure constituting of spindle-shaped cells. IHC with antibody against the latency-associated nuclear antigen (LANA), an obligate molecule for KSHV persistent infection, showed that KSHV latently-infected cells were abundant, many of which were the signature spindle-shaped cells with elongated nuclei (Figs 7B and S6A). To identify endothelial cells, we stained the tumor section with anti-CD31 antibody and found nearly 50% of cells were positive for CD31 expression, indicating their endothelial origin (Fig 7C and S6B). IHC staining with antibody against the Ki-67 proliferation marker identified a high percentage of cells, indicative of their active proliferation (Figs 7D and S6C). These results confirmed that KS tumors are neoplasms of latent KSHV infected cells.

To define the inflammatory nature of the KS tumors, we probed a number of immune components, including T cells, macrophages and dendritic cells. Their presence in KS tumors has been implicated in the prognosis and severity of KS [37]. Antibody against the common CD3 antigen reacted with cells scattered in sectioned tumor (Fig 7E), with regions were highly enriched for CD3-positive cells (S6D Fig). This is likely due to lytic replication of KSHV in a group of highly localized tumor cells. To further probe antigen-presenting cells (APCs) in the tumor, we choose antibody against CD80, a marker for matured APCs, for IHC analysis. This identified some densely packed tumor cells that detected low level of CD80 expression (Fig 7F). Using antibody against a well-defined macrophage marker, IBA-1 [38], we discovered that a significant number of macrophages were present in the highly dense tumor region (Figs 7G and S6E). By contrast, dendritic cells stained with anti-CD11c were less abundant (Fig 7H). Taken together, human KS tumors are extensively infiltrated with immune cells, critical components of organ inflammatory responses.

## Discussion

In this study, we report that introduction of kGPCR into murine γHV68 enables the virus to induce angiogenic lesions in infected mice. Despite that γHV68 encodes its own GPCR homologue, wild-type γHV68 is not sufficient to induce KS-like lesion or malignancies in infected laboratory mouse. In fact, mGPCR failed to activate multiple signaling cascades that are potently up-regulated by kGPCR. Additionally, kGPCR, but not mGPCR, induced tumor formation in a xenograft nude mouse model. One notable difference between these two viral GPCRs is their distinct intracellular localization. While kGPCR primarily resides in TGN, mGPCR appears to localize in the ER compartment, a phenomenon that may underpin the differential signaling capacity. Regulation by cognate ligands at the plasma membrane appears to be shared by both kGPCR and mGPCR [25,27]. It is possible that the post-translational modification indigenous to TGN is a limiting factor for viral GPCR signaling. Events such as glycan modification/editing and tyrosine sulfation occur in the TGN and are relevant to the trafficking and signaling of GPCRs. We previously reported that the N-terminal tyrosine-containing motif of kGPCR was sulfated within the TGN compartment and that sulfation promoted kGPCR signaling and tumorigenesis [39]. Further experiments will be needed to examine these possibilities. Nevertheless, these results demonstrate the fundamental difference between the two closely-related viral GPCR homologues in signaling and tumorigenesis, prompting us to develop a rodent tumor model utilizing a recombinant γHV68 carrying kGPCR.

Multiple transgenic mouse models have been developed in which kGPCR, when expressed in a ubiquitous or endothelium-specific manner, is sufficient to induce tumor lesions [14,16,33]. These mouse tumors recapitulate key pathological features of human Kaposi’s sarcoma, including the spindle-shaped tumor cells and angiogenic vasculature. Though these models excel in tumorigenesis induced by single gene product, they lack the viral infection component. With a recombinant γHV68 carrying kGPCR, we now show that infection in mice triggered angiogenic tumors when T cells were muted with cyclosporine A. Interestingly, recombinant γHV68.kGPCR also displayed faster replication kinetics in cultured cells and formed larger plaques, indicating that kGPCR enhances viral lytic replication in vitro. This observation is consistent with previous reports that kGPCR-mediated signaling enhances the lytic replication of KSHV likely via RTA [40,41], although the molecular detail by which kGPCR impinges on viral lytic replication remains to be further explored. Despite that γHV68.kGPCR undergoes more robust lytic replication in vitro than wild-type γHV68, both viruses established similar levels of latency as judged by viral genome frequency and reactivation capacity of splenocytes. This suggests that host immune response dominates viral infection and facilitates a latent infection, agreeing with the observation that γHV68 established similar levels of long-term latency independent of doses and routes of infection [35]. Consistent with this, we observed that the frequency of splenocytes carrying γHV68.kGPCR genome was only slightly increased compared to wild-type γHV68. Conceivably though, this subtle increase in viral persistence in vivo may amplify under conditions of immune-suppression. As such, when T cells were inhibited with cyclosporine A, mice infected with γHV68.kGPCR developed hepatic and subcutaneous tumor lesions, while those infected with wild-type γHV68 displayed no malignancies when experiment was terminated. To date, we had five, out of 15 mice, developed angiogenic tumors within the time frame of one year. We will continue to optimize the recombinant γHV68-infection tumor model. One possibility is to introduce additional KSHV-specific pathogenic factors, which are devoid in the γHV68 genome [18], into the recombinant γHV68.kGPCR.

While KS lesions develop predominantly in the skin, it is not rare to find on the surface of internal organs, including the liver. Hepatic KS has been diagnosed with ultrasound or x-ray imaging, but biopsy has not been available [42–44]. A retrospective autopsy analysis on AIDS patients discovered that the most frequent benign neoplasm was hepatic hemangiomas [45]. Thus, a rodent model of hepatic KS may provide insight into the pathological features and fundamental sarcomagenesis of human hepatic KS, despite that the frequency of hepatic KS is relatively low in our small cohort of experimented mice. In addition to nodule KS-like lesion in the liver, mice infected with γHV68.kGPCR also developed subcutaneous tumor that was infiltrated with various immune cells, critical components in human KS lesion. These cells include erythrocytes, T cells, macrophages and dendritic cells. Among them, macrophage was abundant in tumors compared to the other immune cells, it would be interesting to determine their roles in sarcomagenesis, if any. A previous report also identified macrophages in KS-like tumors derived from Tva mice expressing kGPCR [14]. It is the M2 type macrophage that is implicated in promoting tumor development, while M1 type macrophage inhibits [46]. When mouse lesions were compared to human KS tumors, H&E and IHC analyses revealed the common histopathological features shared by both neoplasms, including slit-like structure, infiltration of diverse immune cells and the abundant CD31+ endothelial cells in the tumor. However, these two types of tumors also differ in the status of T cells, i.e., with scarce T cells present in mouse angiogenic lesion. This likely stems from the application of cyclosporine A to nullify T cells in the mouse infection model. Alternatively, the distinct anatomical locations of these two types of tumors may underpin the difference in pathology. Nevertheless, these results support the conclusion that tumors induced by γHV68.kGPCR infection are angiogenic and pathobiologically relevant to KSHV malignancies, providing a useful model to investigate tumor induction by KSHV genes in the context of a natural course of viral infection.

## Materials and Methods

### Constructs, cell culture and transfection

If not specified otherwise, constructs were derived from pcDNA5/FRT/TO (Invitrogen). kGPCR and mGPCR were amplified by PCR and cloned into pcDNA5/FRT/TO between Bam*HI* and Xho*I*. The HA epitope was inserted upstream of kGPCR and mGPCR coding sequences. HEK293T (ATCC), SV40-immortalized mouse endothelial cells SVEC (kindly provided by Dr. Philip Sharpe, UT Southwestern), NIH 3T12 cells (ATCC) were cultured in DMEM supplemented with 10% (vol/vol) FBS and 100 U penicillin/streptomycin. NIH 3T3 cells (ATCC) were maintained in DMEM supplemented with 10% (vol/vol) newborn calf serum and 100 U penicillin/streptomycin. Transfection was performed with DNA-calcium phosphate precipitation. Stable cell lines were established using lentivirus transduction and selected with puromycin (1 μg/mL).

### Luciferase reporter assay

HEK293T cells were plated one day before transient transfection with 50 ng of the plasmid expressing firefly luciferase (under the control of response elements of NF-κB, NFAT, and AP-1 transcription factors) and 100 ng of the plasmid expressing β–galactosidase (driven by a house keeping glucophosphokinase promoter). Each transfection was balanced with an empty vector pcDNA5/FRT/TO. Thirty hours after transfection, cells were harvested and lysed in passive lysis buffer (Promega) on ice. After centrifugation, supernatant was used to measure luciferase and β–galactosidase activity according to the manufacturer’s instruction.

### Immunoblotting

Cells were collected, rinsed with ice-cold PBS and lysed in NP-40 buffer (50 mM Tris-HCl [pH 7.4], 150 mM NaCl, 1% NP-40, 5 mM EDTA) supplemented with a protease inhibitor cocktail (Roche), and sonicated for 3 times on ice. Whole cell lysates were denatured and resolved by SDS-PAGE. Proteins were transferred to nitrocellulose membrane and blocked in PBS-T containing 3% non-fat milk for one hour at room temperature. Membrane was incubated with primary antibodies overnight at 4°C. Blots were washed extensively and incubated with corresponding IRDye800 conjugated secondary antibodies (Licor) at 1:5000 for one hour at room temperature. Proteins were visualized using Odyssey infrared imaging system (Licor).

To generate rabbit antibody against thymidine kinase (TK or ORF21), the N-terminal region (amino acid 1–300) was cloned into pGEX-4T-1. Expression and purification of GST fusion proteins were carried out as previously described [47,48] Purified proteins were used to immunize rabbits and whole serum was used to probe TK expression from γHV68-infected cells, along with pre-immune serum as a control. Anti-RTA antibody was described previously [49]. Rabbit sera against vGAT (ORF75c) and mLANA (ORF73) were described previously [50,51]. The antibodies were affinity purified using GST fusion antigens.

### Immunofluorescence and immunohistochemistry analysis

Immunofluorescence and immunohistochemistry experiments were carried out as previously described [30,52,53]. Cells were fixed with 4% (vol/vol) paraformaldehyde for 20 min and permeabilized with 0.4% (vol/vol) Triton X-100 for 20 min. For cell surface staining, Triton X-100 permeabilization was omitted from the procedures. Goat serum (10%) was used to block for one hour at room temperature. Cells were incubated with monoclonal anti-HA (Covance), anti-PDI (Stressgene), anti-TGN46 (Abcam), anti-vGAT, anti-mLANA antibodies overnight at 4°C. After extensive washing, cells were incubated with corresponding secondary antibodies for one hour at room temperature. DAPI was stained before mounted onto microscope slides. Cells were analyzed with a Nikon E800M microscope.

Mouse or human tissues were fixed in 10% neutral buffered formalin (Sigma) overnight at room temperature. Tissues were then dehydrated, embedded in paraffin, and cut into 3-μm sections. After antigen retrieval, tissue sections were subject to H&E staining and immunohistochemical staining with antibodies against HA epitope (OriGene), CD31 (Abcam), CD3 (Abcam), CD11c (Abcam), Ki67 (Abcam), CD80 (Abcam), IBA-1 (Abcam), rabbit or mouse ABC staining system (Santa Cruz), and DAB substrate kit (Vector laboratories).

### Generating γHV68.kGPCR recombinant virus

The γHV68.kGPCR virus was generated from the parental wild-type γHV68.ORF73bla marker virus bacterial artificial chromosome (BACmid) backbone [34] using allelic exchange, as previously described [54]. Briefly, full-length kGPCR was cloned from KSHV genomic DNA and ligated in place of full-length mGPCR in a pGS284 allelic exchange vector [54] carrying wild-type mGPCR and flanking homology arms. Allelic exchange was performed using pGS284.kGPCR in S17λpir and γHV68.ORF73bla BACmid GS500 *Escherichia coli*, as previously described [54]. Following positive and negative selection, diagnostic restriction digests were performed on multiple clones to determine the integrity of the viral genome, and the region of interest was directly sequenced to confirm correct insertion. A single validated BACmid clone was transfected into NIH 3T3 murine fibroblasts to generate high-titer viral stocks, and resulting virus stock was serially passaged in NIH 3T3 cells stably expressing Cre recombinase, resulting in the removal of the *loxP*-flanked BAC sequence and the generation of the γHV68.kGPCR virus. γHV68.rev was generated by recombination of wild-type mGPCR sequence into the identical γHV68.kGPCR BACmid clone.

### Limiting-dilution nested PCR (LD-PCR) detection of γHV68 genome-positive cells

The frequency of splenocytes harboring γHV68 genome was measured by LD-PCR as previously described [35]. Briefly, mouse spleens were homogenized, re-suspended in isotonic buffer and subjected to 3-fold serial dilutions (from 10^4^ to 41 cells/well) in a background of uninfected RAW 264.7 cells, with a total of 104 cells per well. Twelve replicates were plated for each cell dilution. After being plated, cells were subjected to lysis by proteinase K at 56°C for 8 hours. Following inactivating the enzyme for 30 minutes at 85°C, samples were subjected to nested PCR using primers specific for γHV68 ORF72. Reaction products were separated using 2.5% UltraPure agarose (Invitrogen) gels and visualized by ethidium bromide staining.

### γHV68 multi-step growth curve measurement

To measure ex vivo viral growth kinetics, NIH 3T3 cells were infected with γHV68.WT, γHV68.kGPCR or γHV68.Rev at a multiplicity of infection (MOI) of 0.05. Cells and supernatant at different time points were collected and viral titer was determined by plaque assay. To do that, samples were serially diluted and plated onto NIH 3T3 in 24 wells in replicate. Infection was carried out at 37°C for two hours with frequent rocking every 15 min. Cells were then overlaid with DMEM containing 2% NCS and 2% methylcellulose. Cells were incubated for 5 days at 37°C and eventually stained with 0.33% neutral red. Plaques were visualized under microscope and all titers were determined in parallel.

### Ethics statement

All animal experiments were carried out according to the National Institutes of Health principles of laboratory animal care and approved by the University of Southern California Institutional Animal Care and Use Committee (IACUC) with permit number A0372.

### Mice and tumor formation in vivo

The xenograft experiments were performed as described previously [30,39,53]. Briefly, 1×10^5^ stable SVEC cells expressing mGPCR or kGPCR, along with 1×10^5^ bystander SVEC cells were subcutaneously injected into the flanks of 6- to 8-week-old athymic (*nu/nu*) nude mice (Jackson Laboratory). Tumor formation was monitored twice every week and tumors were weighed when mice were euthanized. For infection with recombinant γHV68, 6- to 8-week-old Balb/c mice were intraperitoneally injected with 10^5^ PFU γHV68.WT or γHV68.kGPCR. A week after inoculation, mice were treated with 10 mg/kg cyclosporine A via intraperitoneal injection twice per week. Body weight was monitored every month. All animal experiments were performed according to the National Institutes of Health principles of laboratory animal care and approved by the University of Southern California Institutional Animal Care and Use Committee (IACUC).

### Statistical analysis

Statistical analyses were performed using an unpaired, two-tailed Student’s *t*-test. P values of less than 0.05 were considered to be statistically significant. Mice experiment data were analyzed using GraphPad Prism software (GraphPad Software. Statistically significant p values are indicated by asterisks: *p < 0.05; **p < 0.01; ***p < 0.001.

## Supporting Information

S1 Methods Viral genome analysis. In total, 19,125,847 reads mapped to the reference genome of murine gamma herpesvirus 68 (γHV68) were used to assemble the genome of BAC-γHV68 with assembler Velvet. The maximum N50 could be achieved as 70899 and the mean coverage depth was 1062 when *kmer* was used as 75. The finally assembled genome was incomplete and only covered 93.9% of the MHV68 reference genome, which consisted of five contigs as the length of 70899, 26203, 11350, 3560 and 107 bp, separately. The variants of BAC-γHV68.kGPCR were called based on the five contigs of BAC-γHV68 by using reads aligner BWA and variants caller GATK. In comparison of the reference contigs of BAC-γHV68, only three variants had been discovered, which resided in the topmost two contigs (70899 and 26203). The quality value *QUAL* and the depth of coverage of all three variants were very high.
(DOCX)

S1 Fig Characterize recombinant γHV68. (A) GEMBO DNA carrying γHV68 wild-type (wt), γHV68.kGPCR and γHV68 revertant (γHV68.Rev) were digested with Bam*HI* and analyzed by agarose gel electrophoresis. Arrow indicates the fragment that was cleaved by Bam*HI* within the kGPCR gene. (B) The kGPCR locus was PCR amplified and sequenced. Region flanking the start codon was shown for all three recombinant γHV68. (C) Summary of the point mutations within the γHV68.kGPCR in comparison to γHV68.wt and reference γHV68 genome (accession number: U97553). (D) NIH 3T3 fibroblasts were infected with wild-type γHV68 or γHV68.kGPCR (MOI = 1). Cells were harvested at indicated time points and total RNA was extracted. Total RNA was used to prepare cDNA that was analyzed by real-time PCR with primers specific for indicated genes. All p values were calculated in reference to the control γHV68 wild-type group, *p < 0.05; **p < 0.01; ***p < 0.001.
(TIF)

S2 Fig Persistent infection of wild-type γHV68 and γHV68.kGPCR. Mouse splenocytes were harvested at 16 and 45 days post-infection. Splenocytes were lysed mechanically and incubated with MEFs to allow plaque formation, which assesses preformed virion particles (disrupted).
(TIF)

S3 Fig Angiogenic lesions induced by recombinant γHV68.kGPCR. Subcutaneous angiogenic tumors were analyzed by immunohistochemistry staining with indicated antibodies. (A) kGPCR expression in angiogenic tumors of the subcutaneous compartment. (B-D) Tumor sections were analyzed by IHC staining with antibodies against CD31 (B), Ki-67 (C) and IBA-1 (D). Scale bars denote 40 μm.
(TIF)

S4 Fig Specificity of antibodies against γHV68 LANA and vGAT. Purified antibodies against LANA (A) and vGAT (C) were analyzed by immunofluorescence staining using mock- and γHV68-infected NIH 3T3 cells. Whole cell lysates of γHV68-infected NIH 3T3 cells were analyzed by immunoblotting with antibodies against LANA (B) and vGAT (D).
(TIF)

S5 Fig Effect of cyclosporine A on lytic replication of recombinant γHV68. NIH 3T3 cells were infected with wild-type γHV68 (A and B) or γHV68.kGPCR (C and D) at MOI of 1, without or with cyclosporine A (0.5 or 1.5 μM). Supernatant and cells were harvested, frozen/thawed three times and centrifuged supernatant was used to determine the titer of recombinant γHV68 by plaque assay.
(TIF)

S6 Fig Immunohistochemistry staining of human Kaposi’s sarcoma. Human Kaposi’s sarcoma lesions were stained with antibodies against the KSHV latency-associated nuclear antigen (LANA) (A), CD31 (B), Ki-67 (C), CD3 (D) and IBA-1 (E). For (D), the boxed region was amplified and shown on the right. Scale bars denote 40 μm.
(TIF)
