# Supplementary material for: Recombinant Murine Gamma Herpesvirus 68 Carrying KSHV G Protein-Coupled Receptor Induces Angiogenic Lesions in Mice
Source: PLoS Pathog. 2015 Jun 24;11(6):e1005001. doi: 10.1371/journal.ppat.1005001 (PMC4479558; doi:10.1371/journal.ppat.1005001)
Supplement: S1 Methods — In total, 19,125,847 reads mapped to the reference genome of murine gamma herpesvirus 68 (γHV68) were used to assemble the genome of BAC-γHV68 with assembler Velvet. The maximum N50 could be achieved as 70899 and the mean coverage depth was 1062 when kmer was used as 75. The finally assembled genome was incomplete and only covered 93.9% of the MHV68 reference genome, which consisted of five contigs as the length of 70899, 26203, 11350, 3560 and 107 bp, separately. The variants of BAC-γHV68.kGPCR were called based on the five contigs of BAC-γHV68 by using reads aligner BWA and variants caller GATK. In comparison of the reference contigs of BAC-γHV68, only three variants had been discovered, which resided in the topmost two contigs (70899 and 26203). The quality value QUAL and the depth of coverage of all three variants were very high. (DOCX) [file ppat.1005001.s001.docx]

**Supplemental Methods**

**Viral genome analysis.** In total, 19,125,847 reads mapped to the reference genome of murine gamma herpesvirus 68 (γHV68) were used to assemble the genome of BAC-γHV68 with assembler Velvet. The maximum N50 could be achieved as 70899 and the mean coverage depth was 1062 when *kmer* was used as 75. The finally assembled genome was incomplete and only covered 93.9% of the MHV68 reference genome, which consisted of five contigs as the length of 70899, 26203, 11350, 3560 and 107 bp, separately. The variants of BAC-γHV68.kGPCR were called based on the five contigs of BAC-γHV68 by using reads aligner BWA and variants caller GATK. In comparison of the reference contigs of BAC-γHV68, only three variants had been discovered, which resided in the topmost two contigs (70899 and 26203). The quality value *QUAL* and the depth of coverage of all three variants were very high.
