# Supplementary figures and images for: Recombinant Murine Gamma Herpesvirus 68 Carrying KSHV G Protein-Coupled Receptor Induces Angiogenic Lesions in Mice
Source: PLoS Pathog. 2015 Jun 24;11(6):e1005001. doi: 10.1371/journal.ppat.1005001 (PMC4479558; doi:10.1371/journal.ppat.1005001)

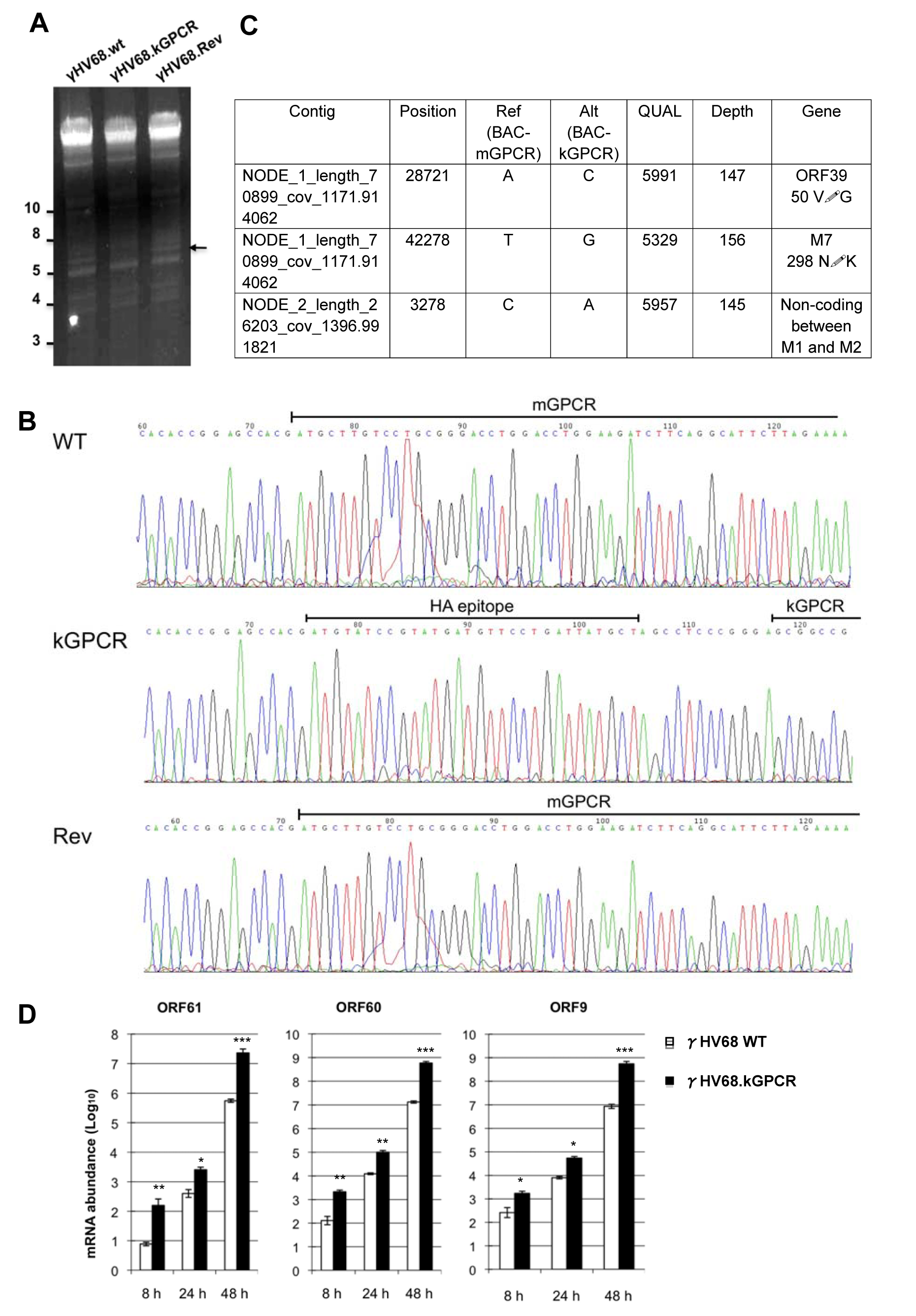

Supplement: S1 Fig — (A) GEMBO DNA carrying γHV68 wild-type (wt), γHV68.kGPCR and γHV68 revertant (γHV68.Rev) were digested with BamHI and analyzed by agarose gel electrophoresis. Arrow indicates the fragment that was cleaved by BamHI within the kGPCR gene. (B) The kGPCR locus was PCR amplified and sequenced. Region flanking the start codon was shown for all three recombinant γHV68. (C) Summary of the point mutations within the γHV68.kGPCR in comparison to γHV68.wt and reference γHV68 genome (accession number: U97553). (D) NIH 3T3 fibroblasts were infected with wild-type γHV68 or γHV68.kGPCR (MOI = 1). Cells were harvested at indicated time points and total RNA was extracted. Total RNA was used to prepare cDNA that was analyzed by real-time PCR with primers specific for indicated genes. All p values were calculated in reference to the control γHV68 wild-type group, *p < 0.05; **p < 0.01; ***p < 0.001. (TIF) [file ppat.1005001.s002.tif]

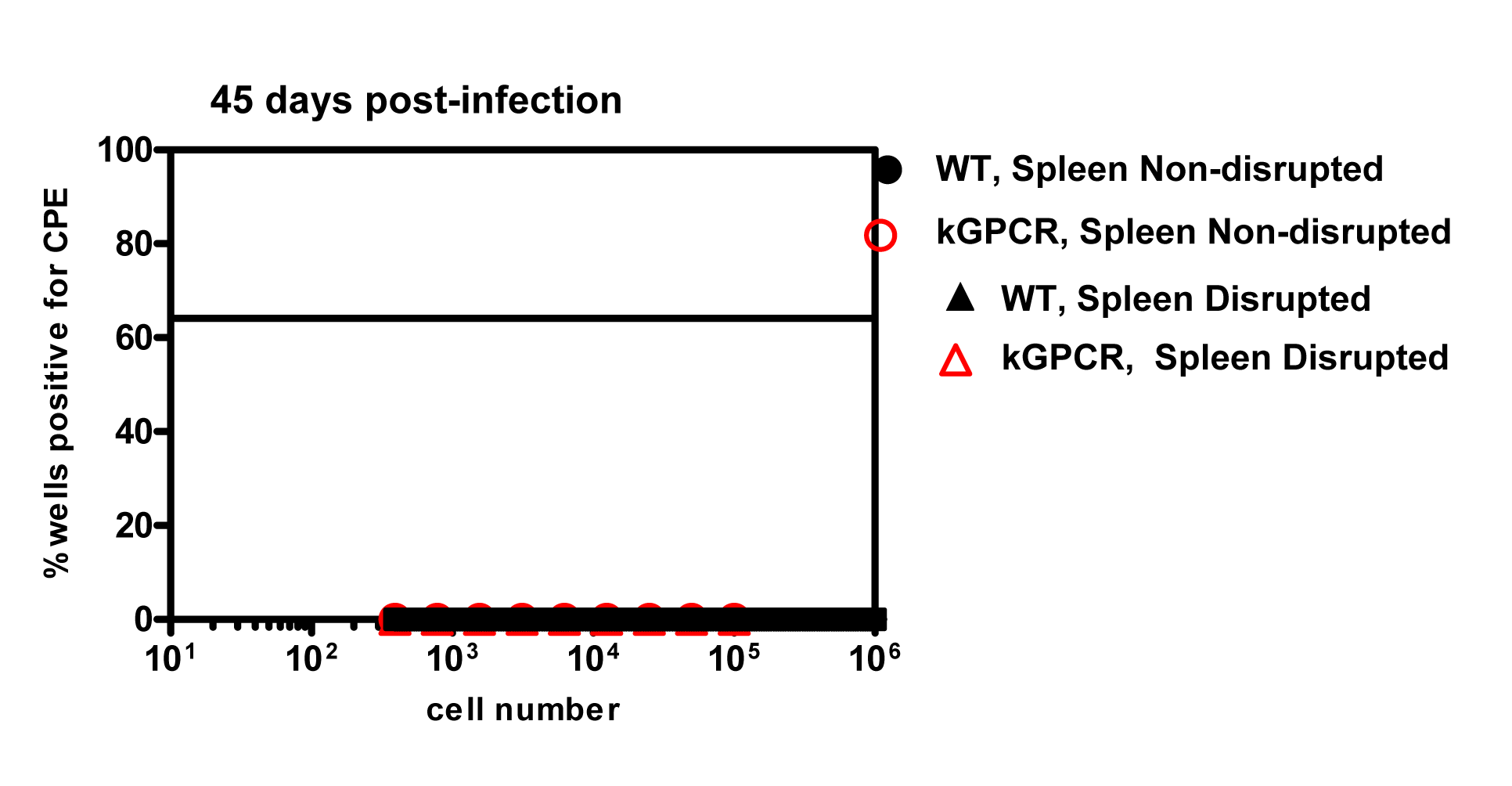

Supplement: S2 Fig — Mouse splenocytes were harvested at 16 and 45 days post-infection. Splenocytes were lysed mechanically and incubated with MEFs to allow plaque formation, which assesses preformed virion particles (disrupted). (TIF) [file ppat.1005001.s003.tif]

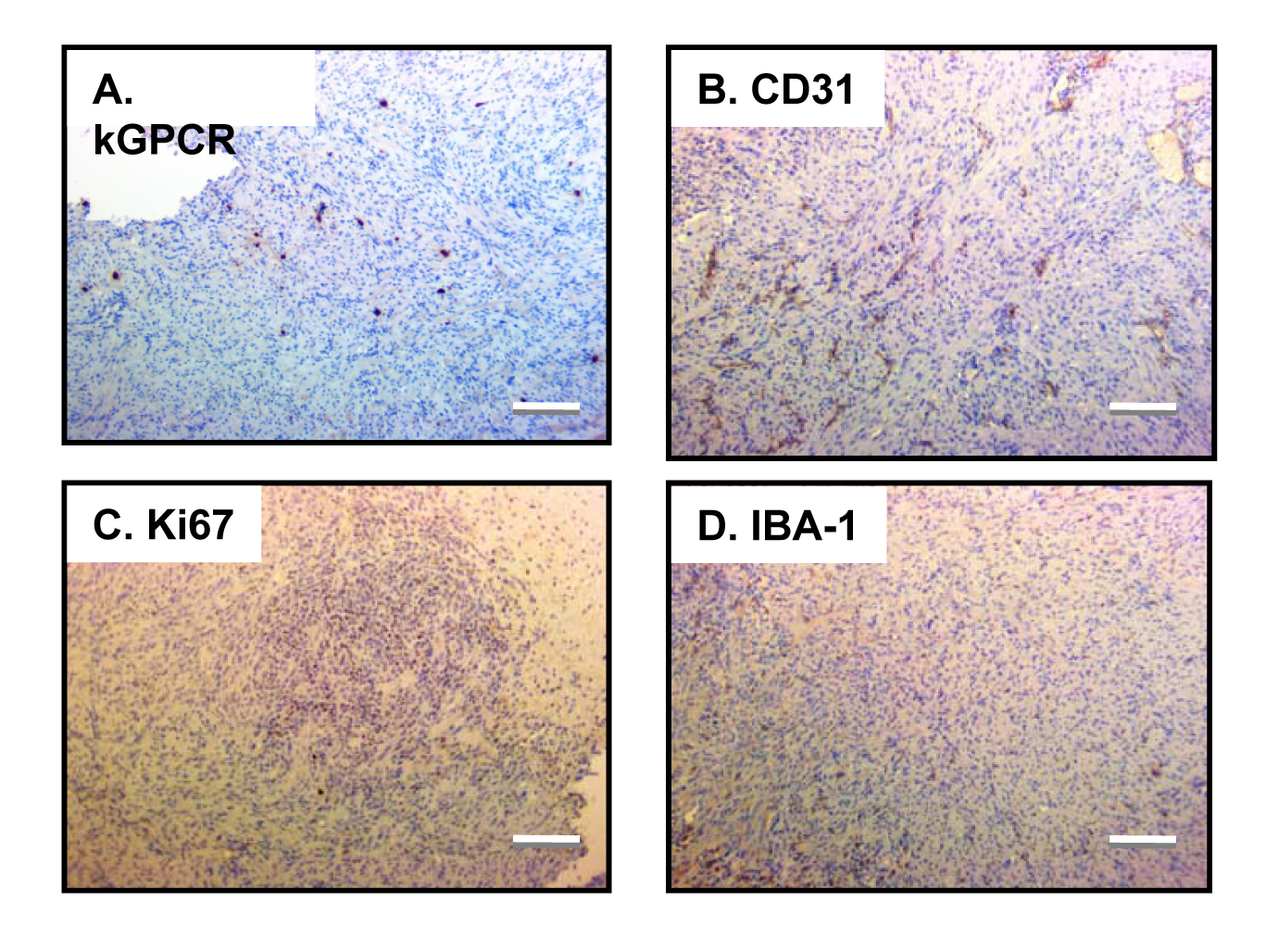

Supplement: S3 Fig — Subcutaneous angiogenic tumors were analyzed by immunohistochemistry staining with indicated antibodies. (A) kGPCR expression in angiogenic tumors of the subcutaneous compartment. (B-D) Tumor sections were analyzed by IHC staining with antibodies against CD31 (B), Ki-67 (C) and IBA-1 (D). Scale bars denote 40 μm. (TIF) [file ppat.1005001.s004.tif]

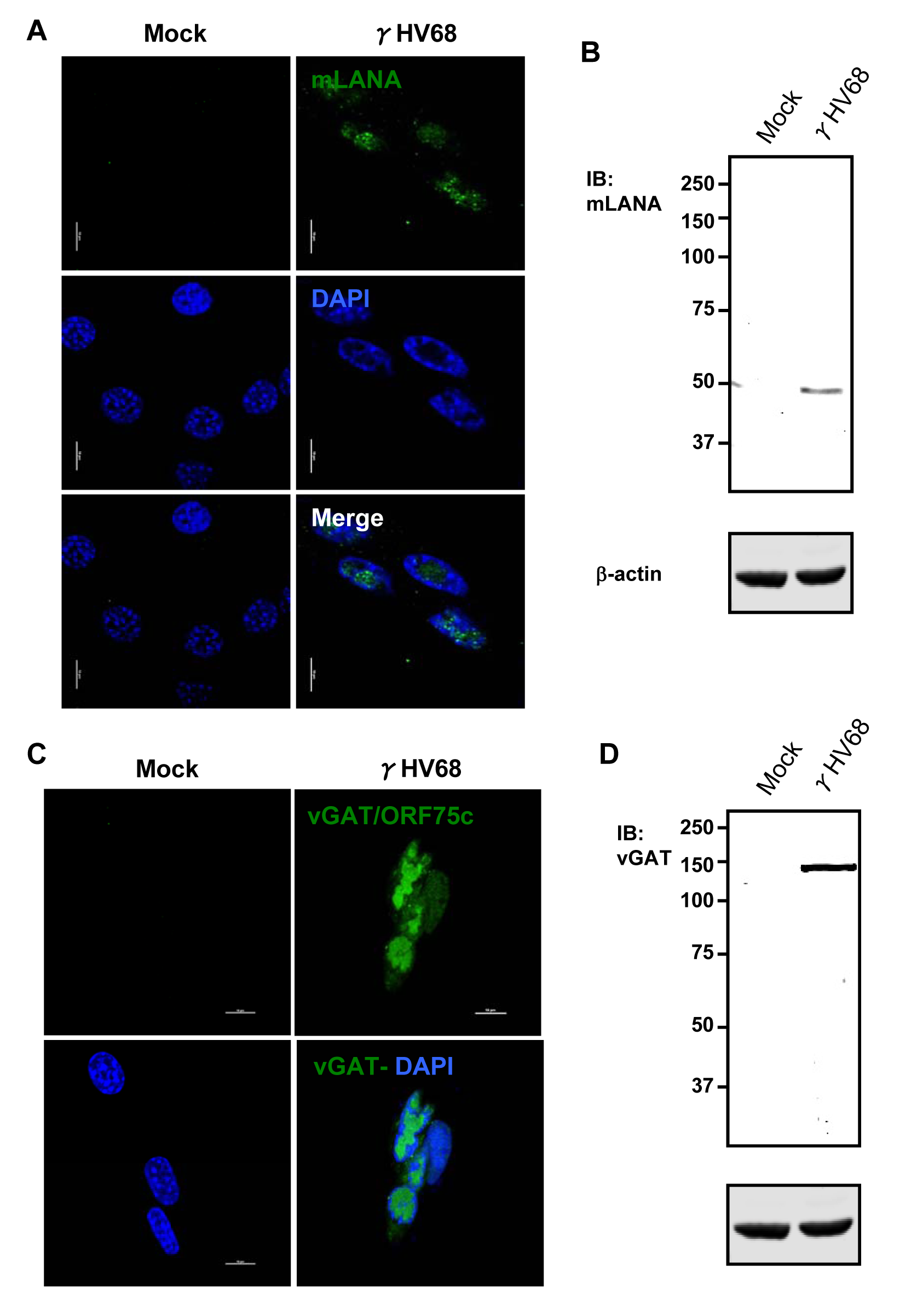

Supplement: S4 Fig — Purified antibodies against LANA (A) and vGAT (C) were analyzed by immunofluorescence staining using mock- and γHV68-infected NIH 3T3 cells. Whole cell lysates of γHV68-infected NIH 3T3 cells were analyzed by immunoblotting with antibodies against LANA (B) and vGAT (D). (TIF) [file ppat.1005001.s005.tif]

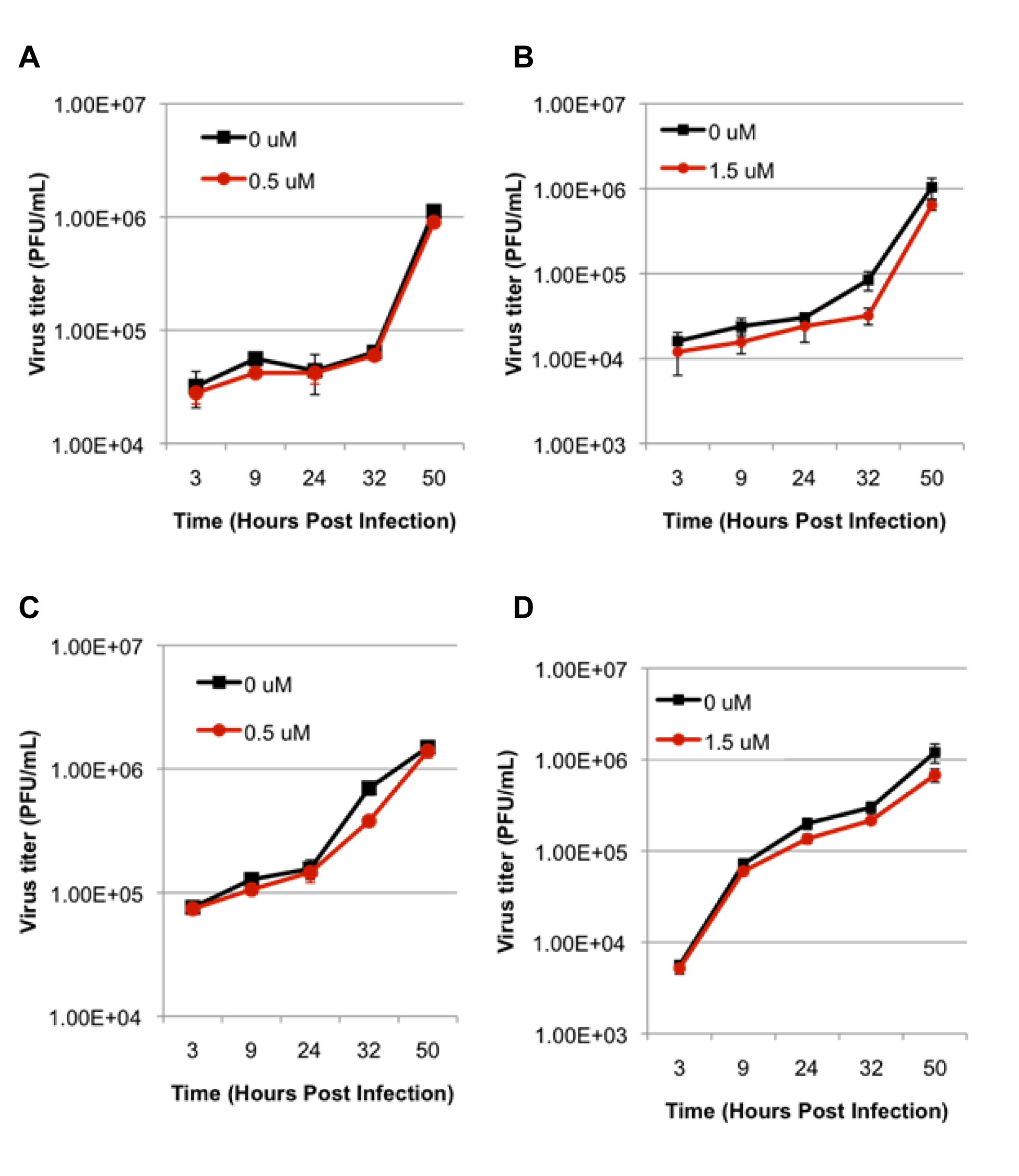

Supplement: S5 Fig — NIH 3T3 cells were infected with wild-type γHV68 (A and B) or γHV68.kGPCR (C and D) at MOI of 1, without or with cyclosporine A (0.5 or 1.5 μM). Supernatant and cells were harvested, frozen/thawed three times and centrifuged supernatant was used to determine the titer of recombinant γHV68 by plaque assay. (TIF) [file ppat.1005001.s006.tif]

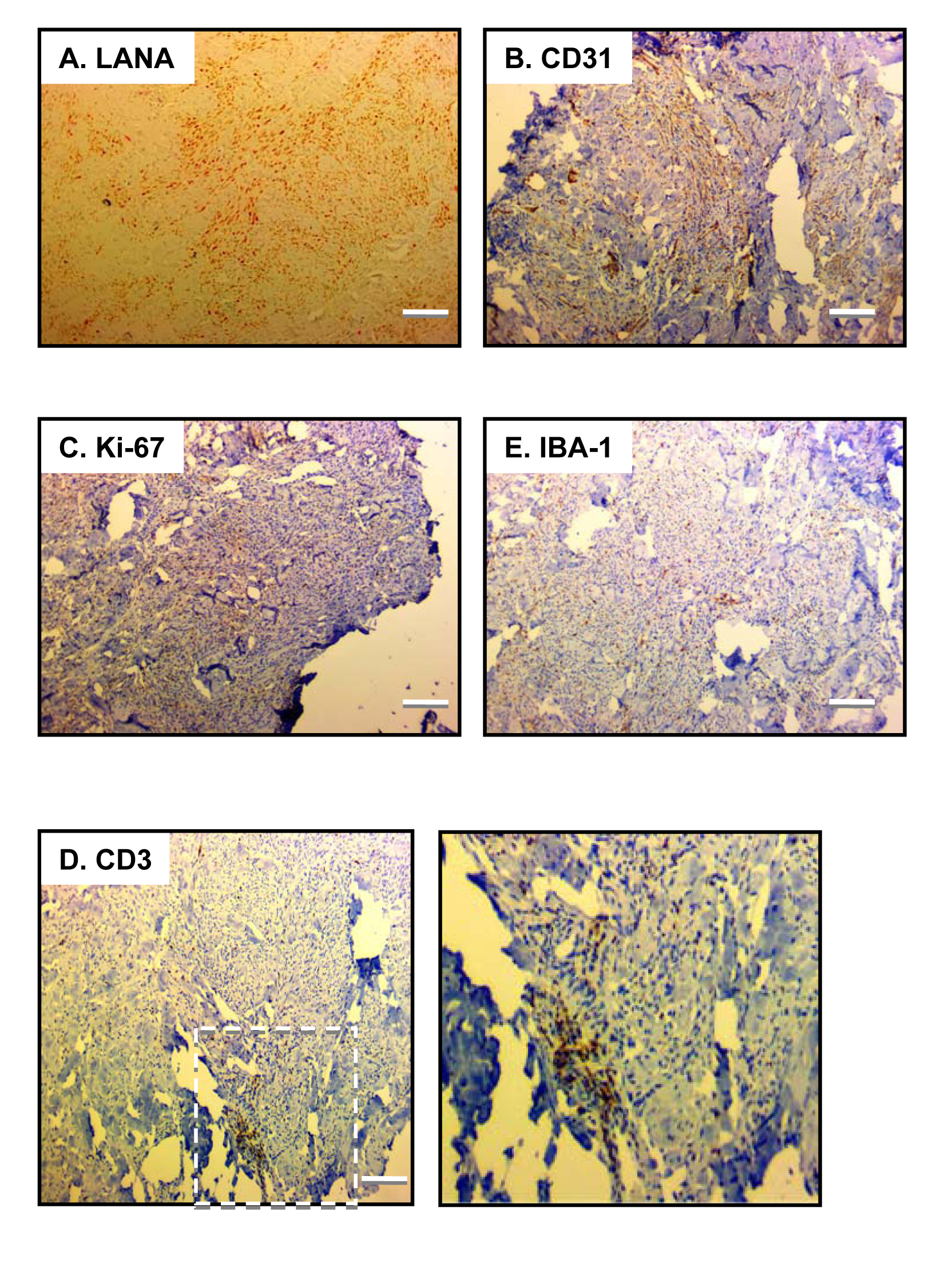

Supplement: S6 Fig — Human Kaposi’s sarcoma lesions were stained with antibodies against the KSHV latency-associated nuclear antigen (LANA) (A), CD31 (B), Ki-67 (C), CD3 (D) and IBA-1 (E). For (D), the boxed region was amplified and shown on the right. Scale bars denote 40 μm. (TIF) [file ppat.1005001.s007.tif]
